# Supplementary material for: Integrating Embeddings from Multiple Protein Language Models to Improve Protein O-GlcNAc Site Prediction
Source: Int J Mol Sci. 2023 Nov 6;24(21):16000. doi: 10.3390/ijms242116000 (PMC10650050; doi:10.3390/ijms242116000)
Supplement: Supplementary file 1 [file ijms-24-16000-s001.zip › ijms-2590688-supplementary.pdf]

Integrating Embeddings from Multiple Protein Language Models to Improve Protein *O*-GlcNAc Site Prediction

Suresh Pokharel [1], Pawel Pratyush [1], Hamid D. Ismail [1], Junfeng Ma [2] and Dukka B. KC [1,*]

[1] Department of Computer Science, Michigan Technological University, Houghton, MI 49931, USA
[2] Department of Oncology, Lombardi Comprehensive Cancer Center,
Georgetown University Medical Center, Georgetown University, Washington, DC 20057, USA
* Correspondence: dbkc@mtu.edu

Table S1 Cost-sensitive learning (CS) and random under-sampling (RUS) based 10-Fold cross validation results of three artificial neural network models trained on Ankh, ESM-2, and ProtT5 embeddings.

Model	Imbalanced Class Handling Method	Mean Accuracy (S.D.)	Mean Sensitivity (S.D.)	Mean Specificity (S.D)	Mean MCC (S.D.)	Mean ROC-AUC (S.D)
Ankh-FFNN	CS	0.8726 (0.0245)	0.5193 (0.0611)	0.8794 (0.0265)	0.1638 (0.0168)	0.7702 (0.0156)
	RUS	0.7141 (0.0078)	0.7236(0.0267)	0.7047 (0.0287)	0.4289 (0.0152)	0.7915 (0.0104)
ESM2-FFNN	CS	0.8801 (0.0367)	0.4590 (0.0862)	0.8947 (0.0404)	0.2043 (0.0393)	0.6923 (0.0364)
	RUS	0.7026 (0.0130)	0.7497 (0.0480)	0.6556 (0.0615)	0.4092 (0.0235)	0.7530 (0.0243)
ProtT5-FFNN	CS	0.8836 (0.0259)	0.5118 (0.0630)	0.8910 (0.0279)	0.1742 (0.0133)	0.7825 (0.0175)
	RUS	0.7152 (0.0068)	0.7352 (0.0073)	0.6952 (0.0149)	0.4308 (0.0134)	0.7872 (0.0105)

Section S1 Cost-sensitive Learning Method

Since the number of positive and negative samples in the training dataset is highly imbalanced, we used a cost-sensitive learning method called class weighting method that uses different costs for classes while training machine learning algorithms. Cost-sensitive learning can be performed using various approaches including data resampling, model training, and ensemble methods. In this work, we explored the class weighting method, where the minority class gets higher weight whereas less weight is given to the majority class while training.

The class weight for each class is calculated as, C_i = the total number of samples / 2 * number of samples in class i. The class weights used for different three models are presented in Table S1.

Table S2 Cost-sensitive learning (CS) and random under-sampling (RUS) based results of Ankh-FFNN, ESM2-FFNN, and ProtT5-FFNN models trained on our training data and tested against our independent test set

Model Name	Imbalanced Class Handling Method	ACC	Sn	Sp	MCC	ROC-AUC
Ankh-FFNN	CS (Cost Sensitive)	0.9176	0.4028	0.9275	0.1674	0.7388
	RUS (Random Under-sampling)	0.7126	0.7122	0.7325	0.1329	0.7962
ESM2-FFNN	CS	0.9277	0.2730	0.9403	0.1193	0.7073
	RUS	0.7059	0.7071	0.6457	0.1050	0.7438
ProtT5-FFNN	CS	0.9189	0.3837	0.9293	0.1606	0.7479
	RUS	0.6999	0.6981	0.8263	0.1334	0.8348
ProtT5+Ankh+ESM2 (Average score level fusion])	CS	0.9562	0.3399	0.9644	0.2087	0.7635
	RUS	0.7495	0.7502	0.7141	0.1447	0.8116
Decision Level Fusion (ProtT5+Ankh+ESM2) (LM-OGlcNAc-Site)	CS	0.9468	0.3495	0.9584	0.1977	0.7635
	RUS	0.8590	0.8648	0.5613	0.1659	0.8116

Table S3 Cost-sensitive learning (CS) and random under-sampling (RUS) based results of Ankh-FFNN, ESM2-FFNN, and ProtT5-FFNN models trained on our training data and tested against O-GlcNAc Pred-II independent test set.

Model Name	Class Imbalance Handling Method	ACC	Sn	Sp	MCC	ROC-AUC
Ankh-FFNN	CS	0.8657	0.6526	0.8688	0.1775	0.8221
	RUS	0.6767	0.7899	0.6751	0.1161	0.8077
ESM2-FFNN	CS	0.8260	0.6442	0.8286	0.1453	0.7953
	RUS	0.5833	0.8123	0.5800	0.1270	0.7717
ProtT5-FFNN	CS	0.8689	0.6778	0.8717	0.1885	0.8351
	RUS	0.6765	0.8347	0.6742	0.1270	0.8348
ProtT5+Ankh+ESM2 (Average score level fusion])	CS	0.8664	0.6946	0.8689	0.1916	0.8457
	RUS	0.6634	0.8263	0.6611	0.1205	0.8320
Decision Level Fusion (ProtT5+Ankh+ESM2) (LM-OGlcNAc-Site)	CS	0.8826	0.6582	0.8858	0.1956	0.8457
	RUS	0.7836	0.7115	0.7846	0.1403	0.8320

Section S2 Performance of LM-OGlcNAc-Site across Disordered and Non-Disordered Regions

We curated a set of 236 proteins distinct from our training set. This set comprised 602 sites unambiguously identified as O-GlcNAc positive, while the remaining 26,982 S/T sites not annotated as positive were considered negative. We utilized a popular protein intrinsic disordered region (IDR) prediction tool, flDPnn, to discern which sites were situated within the disordered region and which were in the non-disordered region. As a result, 2,893 sites (72 positives and 2,821 negatives) were found within the disordered region (IDR), while 24,622 sites (530 positives and 24,092 negatives) were located in the non-disordered region (non-IDR). Subsequently, we segregated the sites based on their regions—disordered and non-disordered—and applied our tool, LM-OGlcNAc, on each dataset separately. Given that the server for O-GlcNAc-Pred II is currently unavailable, we resorted to compare the results with our own test set (which consists of sites in both IDR and non-IDR). The results of LM-OGlcNAc, displayed in Table S4, reveal its performance on disordered regions, non-disordered regions, and mixed (our test set). Interestingly, the results from the IDR closely mirror the performance metrics of the mixed regions (our test set), suggesting that our tool is adept at handling disordered protein regions.

Table S4 Comparative Performance of LM-OGlcNAc-Site across Disordered Regions (IDRs), Non-Disordered Regions (non-IDRs), and Mixed Regions (our test set)

**Region**	**Accuracy**	**Sensitivity**	**Specificity**	**MCC**
IDR	0.8669	0.5139	0.8759	0.1784
Non-IDR	0.8730	0.5717	0.8797	0.1948
Mixed (our test set)	0.8590	0.5613	0.8648	0.1659

Table S5 Class weights calculated to train different models based on the number of negative and positive sites.

**Model**	**Positive**		**Negative**	
	No. of Samples	*Class weights*	*No. of Samples*	*Class weights*
Ankh-model	12644	26.68	647099	0.5095
ESM2-model	12644	14.76	362066*	0.5175
ProtT5-model	12644	26.68	647099	0.5095

* Note: Since ESM-2 has restriction in sequence length, we only considered short sequences (sequence length < 1024) to generate negative training set using ESM-2 embeddings.

Table S6 FFNN Architecture summaries

**Model**	**Model Summary**	**Hyperparameters**
Ankh-<br>FFNN	Layer (type) Output Shape Param #<br>===<br>Dense_1 (Dense) (None, 512) 786944<br>dropout_148 (Dropout) (None, 512) 0<br>Dense_2 (Dense) (None, 128) 65664<br>dropout_149 (Dropout) (None, 128) 0<br>Dense_3 (Dense) (None, 32) 4128<br>dropout_150 (Dropout) (None, 32) 0<br>dense_58 (Dense) (None, 1) 33<br>===<br>Total params: 856,769<br>Trainable params: 856,769<br>Non-trainable params: 0	Learning Rate: 0.001<br>Intermediate Layer Activation Function: ReLU<br>Final Layer Activation Function: Sigmoid<br>Epochs: 100 (Callback Earlystopping)<br>Earlystopping patience: 5<br>Batch Size: 32<br>Optimizer: Adam (adaptive learning rate: 10^{-5})<br>Loss function: binary cross-entropy loss<br>Dropout rates: 0.2
ESM2-<br>FFNN	Layer (type) Output Shape Param #<br>===<br>Dense_1 (Dense) (None, 512) 1311232<br>dropout_212 (Dropout) (None, 512) 0<br>Dense_3 (Dense) (None, 64) 32832<br>dropout_213 (Dropout) (None, 64) 0<br>dense_71 (Dense) (None, 1) 65<br>===<br>Total params: 1,344,129<br>Trainable params: 1,344,129<br>Non-trainable params: 0	Learning Rate: 0.001<br>Intermediate Layer Activation Function: ReLU<br>Final Layer Activation Function: Sigmoid<br>Epochs: 100 (Callback Earlystopping)<br>Earlystopping patience: 5<br>Batch Size: 32<br>Optimizer: Adam (adaptive learning rate: 10^{-5})<br>Loss function: binary cross-entropy loss<br>Dropout rates: 0.2
ProtT5-<br>FFNN	Layer (type) Output Shape Param #<br>===<br>Dense_1 (Dense) (None, 512) 524800<br>dropout_30 (Dropout) (None, 512) 0<br>Dense_2 (Dense) (None, 256) 131328<br>dropout_31 (Dropout) (None, 256) 0<br>Dense_3 (Dense) (None, 128) 32896<br>dropout_32 (Dropout) (None, 128) 0<br>dense_19 (Dense) (None, 1) 129<br>===<br>Total params: 689,153<br>Trainable params: 689,153<br>Non-trainable params: 0	Learning Rate: 0.001<br>Intermediate Layer Activation Function: ReLU<br>Final Layer Activation Function: Sigmoid<br>Epochs: 100 (Callback Earlystopping)<br>Earlystopping patience: 5<br>Batch Size: 32<br>Optimizer: Adam (adaptive learning rate: 10^{-5})<br>Loss function: binary cross-entropy loss<br>Dropout rates: 0.2

Table S7 Description and formula for the performance evaluation metrices (TP = True Positive, TN = True Negative, FP = False Positive, FN = False Negative)

**Metric**	**Description**	**Equation**
Accuracy	Ratio between the number of correctly classified samples and the overall number of samples	$\frac{(\mathrm{TP} + \mathrm{TN})}{(\mathrm{TP} + \mathrm{TN} + \mathrm{FP} + \mathrm{FN})}$
Sensitivity	Sensitivity is also called True Positive Rate (TPR). It is the probability of a positive test, conditioned on the result truly being positive.	$\frac{TP}{TP + FN}$
Specificity	Specificity is also called True Negative Rate (TNR). It is the probability of a negative test, conditioned on the result truly being negative.	$\frac{TN}{TN + FP}$
MCC	Matthew's Correlation Coefficient (MCC) is a reliable performance metric for the imbalanced dataset. Better MCC scores are achieved if the model performs better in all four components of the confusion matrix (i.e., true positives, false negatives, true negatives, and false positives).	$\frac{TP * TN - FP * FN}{(TP + FP)(TP + FN)(TN + FP)(TN + FN)}$
